# Clinician-Created Video Education for Patients With AF: A Randomized Clinical Trial

**DOI:** 10.1001/jamanetworkopen.2023.45308

**Published:** 2023-12-08

**Authors:** Daniel McIntyre, Joshua G. Kovoor, Clara K. Chow, Desi Quintans, Simone Marschner, Stuart Thomas, Pramesh Kovoor, Aravinda Thiagalingam

**Affiliations:** 1 Westmead Applied Research Centre, Faculty of Medicine and Health, University of Sydney, Sydney, Australia; 2 Department of Cardiology, Westmead Hospital, Sydney, Australia; 3 Adelaide University, Adelaide, Australia

## Abstract

**Importance:**

Patient education is a critical aspect of atrial fibrillation (AF) management. However, there is limited time to provide effective patient education during routine care, and resources available online are of variable quality.

**Objective:**

To determine whether clinician-led creation of video-based AF education is feasible and improves knowledge of AF.

**Design, Setting, and Participants:**

This single-center randomized clinical trial was conducted between 2020 and 2022. Outcomes were assessed prior to their clinic visit and 2 and 90 days after the visit by blinded assessors. Participants included adults with AF and congestive heart failure, hypertension, age at least 75 years (doubled), diabetes, prior stroke or transient ischemic attack or thromboembolism (doubled), vascular disease, age 65 to 74 years, and sex category scores of 1 or greater presenting for routine care at publicly funded outpatient cardiology clinics within a tertiary teaching hospital. Individuals too unwell to participate or with limited English were excluded. Data were assessed as intention to treat and analyzed from December 2022 to October 2023.

**Intervention:**

Intervention participants viewed a series of 4 videos designed and narrated by clinicians that aimed to improve understanding of AF pathophysiology, clinical presentation, diagnosis, and management. After viewing the videos, participants received weekly email links to review the videos. The control group received usual care.

**Main Outcomes and Measures:**

The prospectively selected primary outcome was AF knowledge at 90 days, measured by the validated Jessa Atrial Fibrillation Knowledge Questionnaire (JAFKQ).

**Results:**

Among 657 individuals screened, 208 adults with AF were randomized (mean [SD] age, 65.0 [12.2] years; 133 [65.2%] male) and included in analysis. Participants were randomized 1-to-1, with 104 participants in the control group and 104 participants in the video intervention group. At 90 days after the baseline clinic visit, intervention participants were more likely to correctly answer JAFKQ questions than control participants (odds ratio [OR], 1.23 [95% CI, 1.01-1.49]). The difference was greater in participants who remotely accessed videos on 3 or more occasions during the study (OR, 1.46 [95% CI, 1.14-1.88]).

**Conclusions and Relevance:**

In this randomized clinical trial of patients with AF, remotely delivered, clinician-created video education improved medium-term AF knowledge beyond usual care of standard in-clinic education. The improvement demonstrated in this study provides support for the implementation of clinician-created educational resources across the care continuum. Further work is needed to assess for impact on clinical outcomes.

**Trial Registration:**

anzctr.org.au Identifier: ANZCTRN12620000729921

## Key Points

**Question:**

Can clinician-created, video-based education improve knowledge of atrial fibrillation (AF), the most common cardiac arrhythmia?

**Findings:**

In this randomized clinical trial including 204 patients with AF, patients offered video-based education developed by treating clinicians were 23% more likely to correctly answer AF knowledge questions 3 months after their clinic visit, a statistically significant difference.

**Meaning:**

These findings suggest that the clinician-created, video-based education concept could be implemented in other diseases and care settings.

## Teaser

This randomized clinical trial assesses whether clinician-created video-based atrial fibrillation (AF) education is feasible and improves knowledge of AF among patients treated in an outpatient clinic.

## Introduction

Atrial fibrillation (AF) is the most common cardiac arrhythmia, affecting approximately 40 million people worldwide.^1^ Untreated, AF increases stroke risk by 5-fold.^2^ Anticoagulation reduces stroke incidence in patients with high risk but confers a risk of bleeding.^3^ Due to these competing priorities, the decision to treat AF with anticoagulants is complex, requiring risk-benefit consideration and shared decision-making between clinicians and patients.

Poor patient understanding of AF is a significant barrier to achieving these goals. Up to 50% of patients with new AF are unaware of their diagnosis, and more than one-third of patients are unaware of the association of AF with thromboembolism and stroke.^4,5,6^ A patient-centered approach to AF management requires education to facilitate shared decision-making, self-management, and engagement with care. Hence, international guidelines prioritize patient education as a key component of AF management.^7,8^ Despite this, effective education is difficult to achieve within limited clinical contact time, leading to fragmented delivery that may cause patients stress and anxiety with little benefit.^9^

Patients commonly search for disease information on the internet; however, such information is often inaccurate or neglects important components of management. A 2022 study by Luo et al^10^ analyzed 74 online videos on AF, finding 68% were poor quality. Another study by Middeldorp et al^11^ examined 49 AF educational websites and found that only 46% achieved ideal scores on modified patient education materials assessment tools and 26% provided no information on lifestyle modification for AF.^11^

Digitally delivered educational videos created by treating clinicians (hereafter *clinician-created video education*) may bridge this gap and prove a useful adjunct to in-person education by providing patients with evidence-based information from a trusted source in easy-to-understand formats. In a previous nonrandomized study, we delivered clinician-created educational videos to 116 patients with AF and found high satisfaction and improved decision-making, anxiety, and medication adherence.^12^ The Educate-AF randomized clinical trial aimed to examine the impact of clinician-created video education on patient knowledge, medication adherence, and satisfaction with clinical care.

## Methods

This randomized clinical trial was approved by the Western Sydney Local Health District Human Research Ethics Committee. All participants provided written informed consent electronically. The full trial protocol and statistical analysis plan are available in Supplement 1. This report follows the Consolidated Standards of Reporting Trials (CONSORT) reporting guideline for randomized clinical trials.

### Study Design

**Figure 1.**
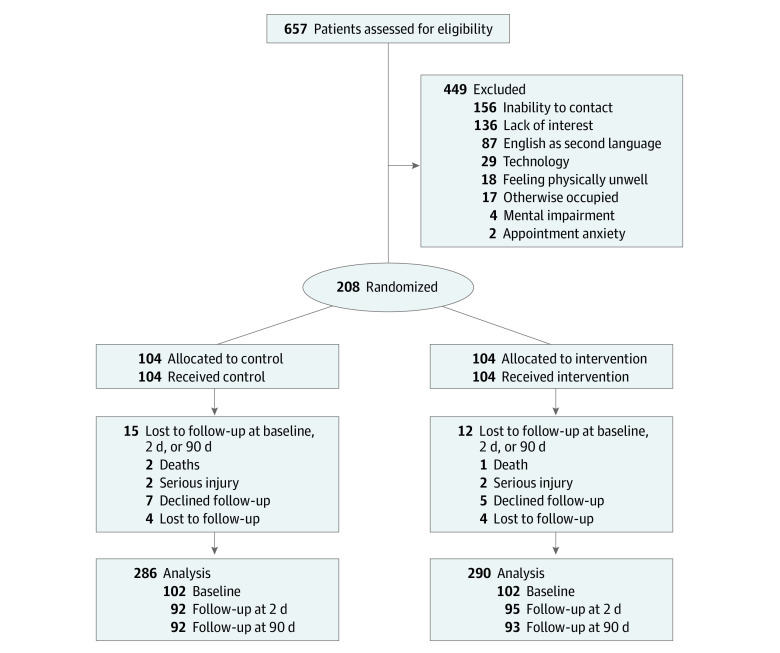
Study Recruitment Flowchart

The Educate-AF study was a single-center, single-blind randomized clinical trial of patients with AF accessing clinical outpatient care within a tertiary teaching hospital in Sydney, Australia (Figure 1). Patients were randomized to receive a series of clinician-created, evidence-based educational videos on AF prior to clinical outpatient contact and weekly for 12 weeks thereafter or usual care.

### Patient Population

Eligible patients were aged 18 years and older with electrocardiography-confirmed AF or flutter of any clinical subtype (paroxysmal, persistent, and permanent); congestive heart failure, hypertension, age 75 years or older (doubled), diabetes, prior stroke or transient ischemic attack or thromboembolism (doubled), vascular disease, age 65 to 74 years, and sex category (CHA_2_DS_2_VASC) score of 1 or greater and/or prescribed anticoagulation therapy; and an active email address or mobile smart phone. Patients were recruited from outpatient clinics within Westmead Hospital, a teaching hospital that serves a diverse catchment (946 000 residents, 46.8% born overseas).^13^ Participants too unwell to participate in surveys, as determined by clinical investigators, or with insufficient English to comprehend intervention content or complete study procedures, as determined by trained study staff, were excluded.

### Recruitment and Consent

Eligible patients were recruited prior to in-person or telehealth outpatient appointments by trained research staff. Screening of all outpatient cardiology clinics was performed to identify eligible participants who were provided with study information and, if agreeable, emailed a link to facilitate participation. This directed patients directly to study consenting documents, where written informed consent was completed via eConsent within REDCap (Vanderbilt University).

### Randomization and Masking

Randomization occurred centrally via a sequence generated within the randomize R library of R statistical software version 3.5.1 (R Project for Statistical Computing) by S.M. Randomization was 1:1 in permuted blocks of 2 and 4 to reduce predictability and ensure balance between study groups. Study staff responsible for recruitment, follow-up outcome assessors, and individuals responsible for statistical analysis were blinded to allocation. Due to the nature of the intervention, it was not possible to blind participants.

### Procedures

The original intervention was designed by J.G.K. and A.T. The development process is described elsewhere.^12^ In brief, following literature and guideline review, investigators formed a syllabus that addressed fundamental concepts of AF pathophysiology, clinical presentation, diagnosis, and management. Original videos displayed a PowerPoint version 16.0 (Microsoft) slideshow presentation (Microsoft) with embedded animation and clinician narration. In the study intervention, videos were updated by D.M. and A.T. within VideoScribe video animation software version 3.6 (Sparkol). D.M. and A.T. learned how to develop content within the video animation software in 1 to 2 weeks. Each video was modified with new scripts developed over 1 week. Videos were then storyboarded, and scripts were provided to narrating clinicians (C.K.C., S.T., AND A.T.) within clinical working hours. Narration was completed in a single take and synced with animation over approximately 2 hours per video by D.M.

The final intervention was a series of 4 videos: (1) “What is AF?” discussing cardiac anatomy, the ECG, and AF risk factors; (2) “Management of Atrial Fibrillation,” addressing the pathophysiology of AF, including the association with stroke risk and pharmacological and procedural management options; (3) “Lifestyle advice,” specifically addressing lifestyle modifications proven to reduce AF burden; and (4) “AF summary,” summarizing previous videos. Patients randomized to the intervention group were automatically directed to watch all 4 videos in order immediately after baseline data collection. The database recorded when videos were opened by participants, facilitating calculation of the number of videos watched. After their clinic appointment, intervention participants were emailed links to review the video series weekly. Ongoing engagement with the intervention was determined by participants and not a requirement of study participation. Videos are freely available elsewhere.^14^ The control group was exposed to usual care, which involved no education beyond that provided during routine clinical care.

### Trial Procedures

Participants underwent assessments at baseline (prior to the first clinic visit), 2 days after their clinic visit, and at 90 days after recruitment. At baseline, we collected demographic information, medication adherence, motivation to maintain medication adherence, and AF knowledge. Race and ethnicity were self-reported by study participants and categorized as Aboriginal or Torres Strait Islander, Asian (including North, East, and South-East Asian), Middle-East and North African, White, and other (including Central and South American, Pacific Islander, Polynesian, and Sub-Saharan African). Race and ethnicity were included in analysis to account for differences in intervention impact among participants of different sociocultural backgrounds. Time of AF diagnosis was not collected; hence, duration of AF could not be calculated. At the postclinic assessment (defined as 2 days postvisit), we collected information on satisfaction with clinical care, motivation to maintain medication adherence, and AF knowledge. At the 90-day follow-up, we collected information on medication adherence, motivation to maintain medication adherence, and AF knowledge. Patient characteristics and outcomes were collected with a combination of medical record review by trained research assistants and participant self-report.

### Study Outcomes

The primary outcome was prospectively defined as the odds of correctly answering each Jessa Atrial Fibrillation Knowledge Questionnaire (JAFKQ) question in the intervention group compared with the control group at 90 days. The JAFKQ is a 16-item questionnaire that addresses general AF knowledge with anticoagulation-specific questions that are different depending on treatment with warfarin or direct oral anticoagulants. This was developed by Desteghe et al^5^ and used for the study with permission from original authors. The full questionnaire can be obtained on request from the Desteghe et al.^5^

Secondary outcomes included medication adherence, measures of patient satisfaction, and JAFKQ score at the 2-day postclinic assessment. Medication adherence was calculated as the frequency of nonadherence in the intervention group compared with the control group based on responses to 3 questions previously validated in the Coronary Artery Risk Development in Young Adults study.^15^ The questions are available in the trial protocol in Supplement 1. Patient satisfaction was assessed with separate measures of satisfaction with clinical care, education, and motivation to maintain medication adherence, each measured on self-report Likert scales (range, 1-7; higher score indicating higher satisfaction or motivation to maintain medication adherence).

### Statistical Analysis

The original intended sample size of 360 was revised to 200 due to recruitment limitations in the context of the COVID-19 pandemic. A sample size of 200 participants (100 participants per group) was calculated to have 90% power (2-sided, type 1 error 5) to detect a difference in 90-day JAFKQ score of 8.95%. This calculation allowed for 15% attrition and assumed an SD of 18, as previously observed.^5^

Due to the nature of the JAFKQ displaying a different number of questions depending on anticoagulant prescription, the primary outcome was analyzed in a log binomial logistic regression model. Age, sex, education, subtype of AF, and baseline JAFKQ score were included as covariates, due to a possible impact on AF knowledge. Resultant odds ratios (ORs) accompanied by 95% CIs describe the odds of a correct JAFKQ answer in intervention compared with control participants. The significance of difference between proportional secondary outcomes (satisfaction with care, education, motivation to maintain medication adherence, and reported medication adherence) was analyzed in a log binomial model adjusting for age, sex, education, subtype of AF, and baseline score where available. All analyses were completed according to principles of intention to treat. An as-treated analysis examining primary and secondary outcomes among highly engaged (watched videos on ≥3 additional occasions), moderately engaged (watched videos on 1-2 additional occasions) and poorly engaged (watched videos on 0 further occasions) participants was also conducted.

Analyses were conducted using R statistical software version 4.1.2 (R Project for Statistical Computing). *P* values were 2-sided, and statistical significance was set at *P* = .05. Data were analyzed from December 2022 to October 2023.

## Results

Between November 18, 2020, and July 18, 2022, 208 participants were recruited from 657 patients screened in cardiology outpatient clinics (Figure 1). Recruitment was stopped when the target sample size was reached. Of 208 participants, 204 (98.1%) completed baseline assessment, 186 (89.4%) completed baseline and 2-day assessment, and 181 (87.0%) had complete data at baseline, 2-day, and 90-day time points (Figure 1). Participants lost to follow-up were more likely male, although they otherwise had similar demographic characteristics to those who completed follow-up (eTable in Supplement 2). Primary outcome analysis included 204 participants (mean [SD] age, 65.0 [12.2] years; 133 [65.2%] male) with complete baseline and 90-day JAFKQ data, including 104 participants randomized to the intervention group and 104 participants randomized to the control group. Questionnaire data were considered incomplete if less than 80% of JAFKQ questions were answered at a given time point. Most participants (114 participants [56.2%]) had paroxysmal AF, with only 13 participants (6.4%) experiencing their first episode of AF. Almost one-fifth of participants (35 participants [17.2%]) had valvular AF. All baseline demographic and medical characteristics were well matched (Table 1). Of 104 intervention participants, 95 (91.3%) watched at least 1 video and 44 (42.3%) were highly engaged (ie, watched videos on ≥3 occasions). There was no significant difference in sex, age, race and ethnicity, educational attainment, or baseline JAFKQ scores between highly engaged participants and the overall cohort.

**Table 1.** Participant Baseline Characteristics

Characteristic	Participants, No. (%)^a^	
	Control (n = 104)	Intervention (n = 104)
Age, mean (SD)	66 (11.2)	64 (13.2)
Sex		
Male	67 (65.7)	66 (64.7)
Female	35 (34.3)	36 (35.3)
Ethnicity		
Aboriginal or Torres Strait Islander	1 (1.0)	0
Asian (North, East, or South-East)	12 (11.8)	11 (10.8)
Middle-Eastern and North African	4 (3.9)	3 (2.9)
White	66 (64.7)	62 (60.8)
Other^b^	19 (18.6)	26 (25.5)
Education: completed year 12	62 (60.8)	57 (55.9)
Videos watched, median (IQR), No.	NA	9 (8-16)
Engagement		
Poor (0 additional sessions)	NA	9 (8.7)
High (3 or more sessions)	NA	44 (42.3)
Type of AF		
First episode	6 (5.9)	7 (6.9)
Paroxysmal	56 (55.4)	58 (56.9)
Permanent	29 (28.7)	32 (31.4)
Predominant flutter	10 (9.9)	5 (4.9)
Valvular AF	15 (14.9)	20 (19.6)
Comorbidities^c^		
Diabetes	21 (20.6)	17 (16.7)
Coronary artery disease	27 (26.5)	25 (24.8)
Hypertension	61 (59.8)	64 (62.7)
Stroke	5 (4.9)	9 (8.8)
Peripheral vascular disease	7 (7.0)	2 (2.0)
Chronic kidney disease	17 (16.7)	12 (11.8)
Current medications, No.		
1-4	39 (38.2)	43 (42.2)
5-8	41 (40.2)	40 (39.2)
>8	22 (21.6)	19 (18.6)
Baseline JAFKQ score, weighted mean (SD)^d^	0.71 (0.18)	0.69 (0.19)

Abbreviations: AF, atrial fibrillation; JAFKQ, Jessa Atrial Fibrillation Knowledge Questionnaire; NA, not applicable.

^a^ Percentages calculated from available responses.

^b^ Includes Central and South American, Pacific Islander, Polynesian, and Sub-Saharan African.

^c^ Medical history was collected by clinical investigators from the electronic medical record.

^d^ Mean scores baseline scores were weighted according to number of questions shown to patients.

**Figure 2.**
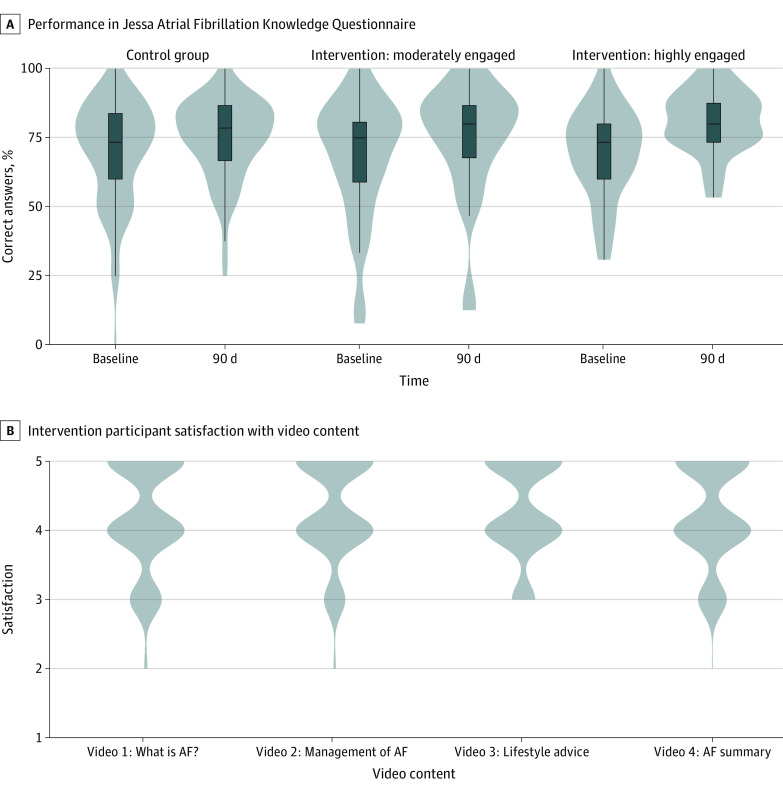
Violin Plot of Knowledge Questionnaire Performance and Satisfaction With Individual Videos The number of participants at each point in the y-axis is indicated by the width of the plot body.

At the 90-day follow-up, intervention participants receiving clinician-created video education had significantly higher odds of correctly answering JAFKQ questions than control participants (OR, 1.23 [95% CI, 1.01-1.49]) (Figure 2). Baseline performance was strongly associated with 90-day performance (OR, 16.13 [95% CI, 9.77-26.80]). Participants who watched videos on 3 or more occasions were more likely to answer the JAFKQ questions correctly (OR, 1.46 [95% CI, 1.14-1.88]). Differences between control and intervention participants were not significant at 2 days after their clinic visit (OR, 1.11 [95% CI, 0.92-1.34]). There was no statistically significant difference between highly engaged intervention participants and control participants at the 2-day follow-up (Table 2).

**Table 2.** Binomial Logistic Regression of Jessa Atrial Fibrillation Knowledge Questionnaire Performance

Intervention group	OR (95% CI)	*P* value
**Performance at 90 d** ^a^		
Overall	1.23 (1.01-1.49)	.04
High engagement^b^	1.46 (1.14-1.88)	.003
**Performance at 2 d** ^a^		
Overall	1.11 (0.92-1.34)	.38
High engagement^b^	1.07 (0.85-1.35)	.58

Abbreviation: OR, odds ratio.

^a^ Adjusted by baseline Jessa Atrial Fibrillation Knowledge Questionnaire performance, atrial fibrillation diagnosis, education level, age, and sex.

^b^ High engagement indicates watching videos on at least 3 separate occasions.

There were no statistically significant differences between intervention and control participants in patient satisfaction with clinical care (OR, 1.15 [95% CI, 0.62-2.16]) or AF education (OR, 1.32 [95% CI, 0.71-2.44]). There remained no statistically significant differences among highly engaged intervention participants compared with control participants (Table 3).

**Table 3.** Satisfaction and Adherence Motivation by Treatment Group

Characteristic	Group, No./total No. (%)		OR (95% CI)	*P* value
	Control	Intervention		
**High satisfaction rating at 2 d**				
Clinician-created video education				
Clinical care	54/92 (58.7)	59/95 (62.1)	1.15 (0.62-2.16)	.66
AF education	43/92 (46.7)	51/95 (53.7)	1.32 (0.71-2.44)	.38
High engagement subgroup^a^				
Clinical care	54/92 (58.7)	26/44 (59.1)	1.02 (0.46-2.27)	>.99
AF education	43/92 (46.7)	20/44 (45.5)	0.95 (0.43-2.07)	>.99
**Motivation to maintain medication adherence at 90 d**				
Clinician-created video education	62/90 (68.9)	66/93 (71.0)	1.04 (0.88-1.23)	.67
High engagement subgroup^a^	62/90(68.9)	30/43 (69.8)	1.08 (0.89-1.31)	.45

Abbreviations: AF, atrial fibrillation; OR, odds ratio.

^a^ High engagement indicates watching videos on at least 3 separate occasions. The high engagement intervention subgroup is compared with the overall control group.

There was also no statistically significant difference between intervention and control groups in motivation to maintain medication adherence at 90 days (relative risk, 1.04 [95% CI, 0.88-1.23]). At study completion, all patients reported adherence to AF-related medication.

## Discussion

This randomized clinical trial found provision of clinician-developed, patient-targeted educational videos prior to and following clinic appointments increased patient-knowledge regarding AF at 3 months compared with usual care. Self-reported medication adherence in the selected population was too high to detect a clinically significant difference. These findings add to a developing evidence base supporting augmentation of outpatient care with opportunistic delivery of high-quality educational resources.^16^ Furthermore, this study provides high-quality evidence that effective and enduring patient education can be developed and delivered by clinicians in a time and resource-efficient fashion. Strengths of this study include its randomized design, successful pilot of remote recruitment and follow-up, use of validated self-reported outcome measures, and implementation within the diverse clinical catchment of Western Sydney Local Health District.

Higher health literacy, facilitated through patient education, is associated with better cardiovascular outcomes.^17^ Traditionally, such education is provided during clinic appointments and ward rounds. However, to ensure retention of clinical information, there is increasing recognition that education must be delivered across the care continuum.^9,18^ The intervention used in this randomized clinical trial was provided prior to an index clinic visit and then offered weekly. There was varied engagement with the continued education, although patients who engaged with weekly emailed videos on 3 or more separate occasions had better knowledge scores. This difference was not significant at 2 days after the index clinic visit, suggesting education provision weeks to months after clinical contact may be more beneficial than immediately after the clinic visit. Other studies have found significant knowledge decay 2 weeks posthospital admission for AF.^19^

There is a large evidence base describing the potential role of video education to improve patient knowledge and clinical outcomes.^20,21^ However, videos available online are not quality controlled or necessarily from reputable sources. A 2018 study by Camm et al^22^ examined 111 patient-focused catheter ablation educational videos, and found that a median of 4 of their 21 essential criteria for high-quality education were met and no videos met all essential criteria. Views and likes were also not associated with video quality.^22^ Clinician-created education can provide patients with evidence-based, high-quality education via a trusted source from within their multidisciplinary treating team. In this study, the education content was developed by physicians; however, the concept of clinician-created content could be broadened to include clinical nurse educators, allied health professionals, and others involved in clinical care.

There are several perceived barriers to wider adoption of clinician-created education. Limited time for clinicians to design and deliver patient-education is a commonly highlighted barrier. In this study, videos were developed over the course of 1 week, within working hours, narrated in a single take, and animated at a time-cost of 2 hours per video. Hence, the time required to develop the intervention was low. Second, clinicians often perceive a skill deficit (ie, assume they are unable to design their own videos) and do not pursue original content development. This leads to increased costs of content development when outsourced to paid third parties, which could have flow on effects in intellectual property ownership of content created. We addressed these issues by purchasing third-party software at low cost. This facilitated design of simple video animations reflective of commonly used sketching by clinicians to illustrate concepts to patients. Investigators D.M. and A.T. self-taught VideoScribe over 1 to 2 weeks, acquiring skills in parallel with video development.

### Limitations

This study has several limitations. The originally intended sample size of 360 participants was not achieved due to recruitment limitations during the COVID-19 pandemic, and we revised the sample size to 200 in consultation with the Western Sydney Local Health District Human Research Ethics Committee. This reduced the accuracy of findings, although the primary outcome was still achieved. The intervention was also consistent among all patients, with little personalization or variation. This kept costs low and facilitated scaled delivery, although it may have limited engagement with protracted delivery. The lack of discrimination among patients for medication adherence measures was disappointing and likely due to limitations of the 3-item scale selected to reduce survey burden. Furthermore, it is possible that contamination occurred in which control participants were exposed to the intervention, diluting intervention effect. Remote content delivery to personal devices reduced this risk. Additionally, time since AF diagnosis and CHA_2_DS_2_VASC score were not measured in this study, which may have interacted with the primary outcome, although all other baseline variables were well matched, suggesting a successful randomization that controlled for measured and unmeasured cofounders. Future studies may consider other measures, such as direct pill count or data linkage to script fulfilment, as accurate measures of medication adherence with little survey burden.

## Conclusions

The findings of this randomized clinical trial suggest that clinician-created educational videos could present a low-cost approach to augment patient-centered cardiovascular care. In this study, short clinician-created, patient-targeted educational videos offered weekly via email improved AF knowledge at 3 months after routine outpatient care. Highly engaged patients were more likely to correctly answer AF knowledge questions. An effect on medication adherence was not identified in this trial but should be investigated in larger studies. This study did not measure any cardiovascular outcomes, but this could be a logical progression of the concept.

## Notes

Supplement 1.
Trial Protocol and Statistical Analysis Plan

Supplement 2. **eTable.** Baseline Demographic Data by Completeness

Supplement 3.
Data Sharing Statement
