# Supplementary material for: Clinician-Created Video Education for Patients With AF: A Randomized Clinical Trial
Source: JAMA Netw Open. 2023 Dec 8;6(12):e2345308. doi: 10.1001/jamanetworkopen.2023.45308 (PMC10709770; doi:10.1001/jamanetworkopen.2023.45308)
Supplement: Supplement 1. — Trial Protocol and Statistical Analysis Plan [file jamanetwopen-e2345308-s001.pdf]

eAppendix 1: Protocol:

1.1 Protocol summary

Atrial fibrillation (AF) is the most common cardiac arrhythmia globally. Patient knowledge of AF and approaches to medical therapy for AF is poor. This study aims to test the utility and scalability of an AF educational program developed by staff at Westmead Hospital.

Hypothesis:

Patients exposed clinician-developed educational videos prior to and post clinic appointment will higher knowledge of AF, medication adherence and satisfaction with clinical care compared to usual care.

Primary Objective:

The primary objective of this study is to assess whether simple, clinician designed educational program can improve knowledge of AF at 90 day follow up compared to usual care.

Design

Randomised clinical trial amongst 200 patients accessing clinical care for atrial fibrillation within Westmead Hospital. Randomisation to intervention and control will be in a 1:1 ratio. Intervention participants (n = 100) will receive a series of 4 clinician-developed educational videos on atrial fibrillation. Control (n = 100) will receive usual care.

Specific Aims:

To examine, among patients with atrial fibrillation accessing clinical care whether a series of clinician-designed educational videos delivered prior to clinic improves:

1. Knowledge of AF
2. Adherence to AF-related medication
3. Satisfaction with clinical care

2. Background:

Atrial fibrillation (AF) is the most common cardiac arrhythmia globally and its prevalence is expected to double by 2050.[1] Patients with AF have an approximately 5-fold increased risk of stroke compared to the general population.[2] Appropriate anticoagulation has been demonstrated to significantly reduce the risk of stroke and mortality in patients with AF.[2] Yet, the decision to anti-coagulate is complex and must be balanced against multiple individual patient factors, especially the concomitant risk of bleeding.

In recognition of the multifaceted and complex nature of AF management, the updated 2018 National Heart Foundation guidelines for the management of AF make a strong recommendation for the use of shared decision making as well as 'targeted patient education throughout the continuum of AF management'.[3] However, the development and implementation of targeted education can be costly and time consuming for both physicians and patients. Due to this, there are limited local AF educational resources available that are appropriate for the patient population at Westmead Hospital.

To address this shortfall, we have developed a series of simple educational videos on AF that cover what AF is, its diagnosis and management. The intervention has been evaluated in an observational, non-randomised study of 104 patients with AF. 96.4% of participants were 'very satisfied' with the videos (5 on 5-point Likert scale). Videos were also rated highly on their potential for reducing peri consultation anxiety (median = 90%), improving decision making ability (median = 90%) and likelihood of following treatment plans (median = 93%). However, we have limited data describing the impact of this intervention on patient understanding of AF, adherence to AF-related treatments and overall satisfaction with clinical experience. Whilst studies have been performed that evaluate the potential for educational interventions to impact AF knowledge and treatment adherence[4,5], we are not aware of any studies that have compared the efficacy of low and high cost educational interventions in the same patient population.

Aim and Objectives: To evaluate the acceptability and utility of a simple clinician designed educational intervention for patients with AF compared to usual care. Specifically, to describe the impact of the intervention on:

- Knowledge of AF at 90 day follow up
- Medication adherence at 90 day follow up
- Satisfaction with clinical care
- Motivation to maintain adherence to AF medication post intervention delivery and at 90 day follow up

3. Research Plan and Methods:

3.1 Experimental design:

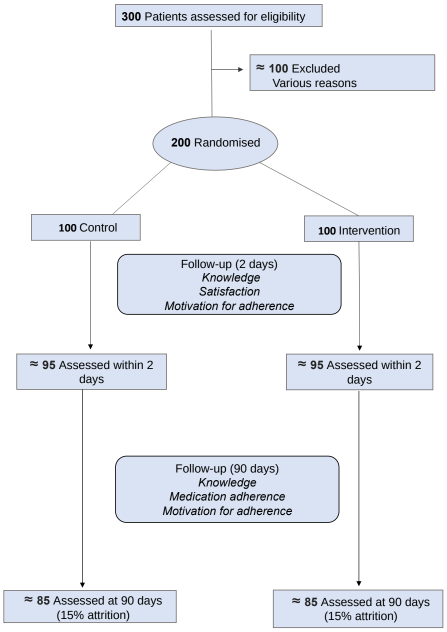

eFigure 1: Participant flow

Overall Study

This is a randomised controlled trial (RCT) of 200 patients who are accessing clinical care at Westmead hospital. Individuals will be randomised in a ratio of 1:1 (intervention : control). Randomisation will occur centrally prior to study implementation to intervention (educational videos) or control (usual care).

3.2 Study population:

The study population will be patients with confirmed AF on an electrocardiogram presenting to Westmead Hospital for clinical care. This includes both inpatients and outpatients.

3.3 Eligibility and recruitment

Inclusion

1. Adults aged 18 years and over
2. Atrial fibrillation or atrial flutter diagnosed on electrocardiogram
3. $CHA_2DS_2VASc > 0$ and/or currently prescribed anticoagulation therapy
4. Have an email address or active mobile number

Exclusion

1. Too unwell (physically or mentally) to participate in surveys

2. Previously allocated to either condition of the study
3. Insufficient English to understand video content or to participate in the audit surveys

Recruitment

Outpatient clinics

Eligible patients will be recruited prior to their physical or telehealth outpatient appointment by trained research staff. Staff will screen clinic lists for all outpatient cardiology clinics and identify patients that meet study inclusion criteria.

These patients will be contacted via telephone by study staff and, if agreeable, will be emailed a link. This link will take participants directly to a study description document, followed by baseline surveys and, depending on intervention allocation, either a message thanking them for their participation or the intervention videos.

If patients are physically attending the hospital for their appointment, they will be offered the study intervention on a study iPad in the clinic waiting room or have the option to participate in the study via their own device prior to the appointment.

Inpatients

Inpatients will be only be approached by study stuff after:

A) The patients treating doctor refers them for recruitment onto the study
B) Westmead hospital's COVID-19 policies allow research staff to approach hospital inpatients for participation in clinical trials

Hospital inpatients, if recruited, will have the option of receiving the intervention on their own device or to have the intervention delivered on a study tablets (iPad) which will be sanitised between patients.

The consent process is detailed in section 9.3.

3.4 Randomisation and blinding:

Overall study

Randomisation will occur centrally via a computer-generated sequence, using the randomise R library of R statistical software (version 3.5.1), before the study commences. Randomisation will be 1:1 and in randomised permuted blocks of 4 and 2 to reduce predictability and ensure a balance between the two arms.

After registration, patients will be allocated a study number. From there-on, their details will be deidentified. Clinic staff and follow-up outcome assessors will be blinded to the condition patients have been allocated to. However, due to the nature of the intervention, participants cannot be blinded to their treatment allocation.

4. Trial intervention:

Control:

Patients randomised to the control arm will view the study description document, complete baseline surveys and then proceed through usual care. In the clinic waiting room this will involve healthcare posters/pamphlets and a small central TV fixed to a low volume. For inpatients, usual care will vary however may include education provided by medical staff on ward rounds, time spent watching TV and ad-hoc education from other hospital staff.

Intervention:

Patients randomised to the intervention arm will view the study description document and complete baseline surveys. Following this, they will be automatically delivered the series of 4 clinician-developed videos.

Intervention participants will then either receive a link to the intervention videos once/week via text/email.

Reviewers can view the video series at the following link:

https://vimeo.com/showcase/7683573

4.1 Intervention development:

The study intervention was developed by investigators AT and JK. The educational audiovisual module was designed to be a web-based combination of four short videos (privately hosted on YouTube) to be viewed sequentially, accompanied by questions gauging patient experience. The module was consolidated using Research Electronic Data Capture (REDCap).[6] Data from the module were collected and coded on REDCap via a secure database.

Investigators decided on an up-to-date, reliable, yet simple syllabus covering the fundamental concepts of the pathophysiology, clinical presentation, diagnosis and management of AF. The four succinct videos within the module encompassed the following topics: (1) 'What is AF?', (2) 'AF Management', (3) 'Stroke risk and anticoagulation', and (4) 'Lifestyle modification'.

The level of patient satisfaction for each individual video, and satisfaction with the videos overall, were recorded on 5-point Likert scales. Further, 0-100 visual analogue scales were used to gauge the patient's response to clinician narration, and the module's utility in improving patient decision making, anxiety around consultation, and long-term treatment adherence. The visual analogue scales and Likert scales were created and combined with the four videos for the audiovisual module using REDCap.

To make the four educational videos within the module, four brief slideshows were created using Microsoft PowerPoint on the laptop, then recorded audio narration simultaneously to annotate the slideshows using the Apple iPad. The videos were subsequently privately hosted on YouTube in order to be consolidated alongside the questions gauging patient experience on REDCap. REDCap then generated a Uniform Resource Locator (URL), also known as a web address, and a Quick Response (QR) code, both of which enabled access to the educational module using any device with Internet access.

All information provided within the module was only included following brief inspection of the latest peer-reviewed literature on the associated topics by the clinician in order to update existing knowledge. The audiovisual module, along with all included images and visual media, was consolidated solely for private use limited to the duration of the study. It was not marketed or sold for commercial purposes.

Following informed consent, patients completed the audiovisual educational module during the otherwise non-utilized waiting time prior to their consultation for AF. Patients accessed the web-based module through either the URL or QR Code generated by REDCap. This was done using either the single Apple iPad that was already owned by the specialist clinic, or mobile smart devices that the patients already owned.

The intervention was then further enhanced by DM and AT utilizing VideoScribe® technology to increase the acceptability of video content.

5. Data Collection and Management:

Baseline, post-clinic and follow up data will either be collected by study staff via phone call and entered remotely into the intervention delivery platform, or entered directly by participants into the delivery platform, RedCap.[7] The study platform will be designed such that it is user friendly across smart phone, tablet and computer. Study staff will be available via phone call to assist patients with data entry and accessing the intervention.

Survey	Baseline	Post-clinic/within 2 days of intervention delivery	90-day follow up
Baseline demographic information	X		
Satisfaction with care (7 – point Likert scale)		X	
Medication adherence	X		X
Motivation to maintain adherence to AF therapy (7-point Likert scale)	X	X	X

AF Knowledge (Jessa AF Knowledge Questionnaire)	X	X	X

eTable 1: Outline of data collection process

Data will be managed in the RedCap® system during the trial with programmed range checks and sense checks. Physical source data for the end of clinic survey will be stored in a locked office accessible only by card. Primary outcome data will be monitored against source data. At study completion data will be exported for analysis. Exported data files will be stored in secure password-protected servers at the University of Sydney in compliance with ethical commitments. Investigators responsible for data analysis will not be involved in collecting identifiable participant information.

Baseline demographic information:

Baseline demographic collection will include:

- Gender
- Age
- Ethnicity
- Educational level
- AF subtype
    - Atrial fibrillation or atrial flutter
    - Non-valvular or valvular
    - First AF episode or paroxysmal ongoing AF or persistent AF or permanent AF or predominant atrial flutter
    - Time since AF diagnosis
- Medical history (diabetes, hypertension, coronary artery disease, stroke, peripheral vascular disease, hyperlipidemia, chronic kidney disease)
- Current medications

AF subtype, medical history and current medications will be collected separately from hospital records by study staff. All other information will be collected in the baseline study assessment form.

Jessa Atrial Fibrillation Knowledge Questionnaire:

The Jessa Atrial Fibrillation Knowledge Questionnaire (JAFKQ) has been developed by Deteghe et al (2016).(6) The questionnaire will be delivered to participants such that:

- All participants answer the first 8 questions (1.1 – 1.8) on general AF knowledge
- ONLY participants on anticoagulation answer questions on anticoagulation (2.1 – 2.5)
- ONLY patients on Warfarin answer questions 3.1-3.3
- ONLY patient on novel oral anticoagulation (NOAC) answer questions 3.4-3.6

Medication adherence:

To assess medication adherence, we will utilise 3 questions adapted for from the Coronary Artery Risk Development in Young Adults (CARDIA) study.[8] This series of questions has previously been used to assess adherence to hypertensive medications in patients with coronary artery disease,[9] and since in a large scale cross sectional study of patients with atrial fibrillation.[10] The questions are:

1) **Specific to your atrial fibrillation medication, in the past month, how often did you take your medications as the doctor prescribed?**
    a. All of the time (100%)
    b. Nearly all of the time (90%)
    c. Most of the time (75%)
    d. About half the time (50%)
    e. Less than half the time (<50%)
    f. No atrial fibrillation medication

2) **Specific to your atrial fibrillation medication, in the past month, how often did you forget to take one or more of your prescribed medications?**
    a. Never

b. Once
c. 2-3 times
d. Once per week
e. Several times per week
f. Nearly every day
g. No atrial fibrillation medicaiton

3) **Specific to your atrial fibrillation medication, in the past month, how often did you decide to skip one or more of your prescribed medications**
   a. Never
   b. Once
   c. 2-3 times
   d. Once per week
   e. Several times per week
   f. Nearly every day
   g. No atrial fibrillation medicaiton

Non-adherence will be defined as per previous studies:
1. Response to question 1 of "Most of the time (75%) or less
2. Response to question 2 of "once per week" or more
3. Response to question 3 of "once per week" or more

6. Assessment of outcomes:
Primary Outcome:
The primary outcome will be the difference in participant percentage score on the Jessa Atrial Fibrillation Knowledge Questionnaire (JAFKQ)[11] in the intervention arm with reinforcement compared to the control at 90 day follow up.

Secondary outcomes assessed at end of clinic (outpatients)/within 2 days (inpatients) will include atrial fibrillation knowledge (JAFKQ)[11], satisfaction with clinical care (7-point Likert scale) and motivation to maintain adherence to anticoagulation therapy (7-point Likert scale). Medication adherence will be measured at 90 day follow up (see etable 1).

7. Statistical considerations and Analysis:
Analysis will follow intention to treat principles with all participants analysed in the arm they have been randomised to. P-values of < 0.05 will be considered significant. Primary outcome analysis will use a two-tailed t-test to assess the difference between mean participant JAFKQ scores (max score = 100). Analysis will also be performed utilizing an Analysis of Covariance (ANCOVA), with baseline JAFKQ scores and education level incorporated into the model as covariates. A sample size of 200 participants (100 per arm) would, allowing for 15% attrition and assuming a standard deviation of 18 as described in other studies,[11] have 90% power (2 sided, type 1 error of 5%) to detect an difference in 90-day AF knowledge of 8.95% between participants in the intervention and control arms of the study, which we consider a significant improvement in knowledge. Previous assessment of the JAFKQ has indicated participant scores 1 month post-test with no educational intervention are unchanged (60.4 [SD = 18.6] vs 62.3 [SD = 18.5]; p = 0.551).[11]

For secondary analysis of satisfaction with clinical care, patient responses will be dichotomised into 'satisfied' ($\geq$ 5/7) vs 'unsatisfied'($\leq$ 3/7). For medication adherence, patients will be classified as "adherent" or "not adherent as per page 11-12. Proportions in intervention vs control arms will be compared using a chi-squared test. Relative risk will be calculated using a log binomial unadjusted and adjusted for total time in clinic (for outpatients) and number of medications. Allowing for 15% attrition and assuming 60% satisfaction/medium or greater adherence in the control groups, a sample size of 200 (100 per arm) would have 90% power to detect a relative risk difference in satisfaction and adherence between control and intervention groups of 1.37 (that is, an increase from 60.0% to 82.2%).

8. Study significance:

Patients accessing public hospital services are rarely provided with engaging material to complement clinical care. Especially during the current COVID crisis, patients are isolated and lack engagement from their health providers.

The intervention assessed in this study, if validated, offers a simple, cost effective way to improve patient health and health literacy. Improved patient health literacy has been linked to better health outcomes in populations of patients with cardiovascular disease,[12] and the potential for better medication adherence could see a direct translation from this study into better health outcomes.

Making use of time that is often wasted to improve patient experience and health is of high interest to health system administrators in both the private and public sectors.

9. Safety

9.1 Data and Safety Monitoring Board (DSMB)

Watching videos is safe and does not cause harm. A DSMB will not be required.

9.2 Adverse event definitions and reporting

There are no anticipated adverse effects from the study intervention.

10. Ethics and Dissemination

10.1 Research and ethics approval

We will seek ethical approval from HREC Westmead Hospital.

10.2 Protocol Amendments

Protocol modifications that could impact the conducting of the study (ie changes to the objectives, study design, subject population, study procedures) must be approved by the ethics committee, and regulatory authorities where required. It is expected that investigators take immediate action that may be required for subject safety, even if this is not in accordance with the protocol. Regulatory authorities and ethics committees should be notified of this within 72 hours.

10.3 Consent

The study consenting documents are displayed in appendix 1. Potential participants will only be included in the study after reading and acknowledging the participant information form. This form outlines the pertinent details of the study to potential participants, including:

- Randomisation between intervention and control (1:1)
- Storage of data and data security/de-identification
- Access to patient data relevant to the study
- Right to receive treatment regardless of participation
- Right to withdraw from the study
- Consent to follow up phone contact form study coordinators at 90 days

Participants will be provided the study participant information form and contact details of relevant HREC staff and study investigators.

Investigators have developed the study information form and consent process such that pertinent study details can be communicated to participants in a way that respects their time whilst recognizing their right to understand the research they are agreeing to participate in and provide informed consent.

Study staff will also explain the details of the study and be available to provide additional information about the study to participants who request. All participants are free to withdraw from the study at any time. If withdrawal occurs, relevant study staff will not collect additional data, although data already collected will be retained to ensure the results of the research project can be measured properly and to comply with law. Data collected by the sponsor up to the time of withdrawal will form part of the research project results. If a patient doesn't want this to occur, they must tell the research team prior to joining the research project.

10.4 Confidentiality

All personal information in the central database will be de-identified. Participant information sheets and survey responses will be stored in a locked office in the Westmead Applied Research Centre only accessible by card. Data entered via study tablets will be stored in the secure RedCap server. The information will be re-identifiable for the research assistant that will be making 90 day follow up calls to participants.

10.5 Post trial care

All participants will be sent links to the videos to view in their own time after the 90 day follow up period.

10.6 Declaration of Interests

There are no competing interests from anyone in the study group.

eConsent Form

Resize font:
⊞ | ⊟

THE UNIVERSITY OF
SYDNEY
—
Westmead Applied
Research Centre

Informed Consent

Before we begin, it is important that:

1. We explain the details of this study to you

2. You understand all study procedures including consent, withdrawal, and what will be done with your information.

Please read these sections carefully. There are staff available that can answer any questions you have.

Participant Information Sheet

Interventional Study - *Adult providing own consent*

Title	*EDUCATE-AF*
Principal Investigator	*Dr Aravinda Thiagalingam*
Location	*Westmead Hospital*

Part 1 What does my participation involve?

1 Introduction
This research project is testing a new method of delivering education to patients with atrial fibrillation (an abnormal heart rhythm). Please read the information on this Participant Information Sheet carefully and ask questions about anything that you don't understand or want to know more about. Participation in this research is voluntary, and you will receive the best possible care whether or not you take part.

2 What is the purpose of this research?
The purpose of this research is to test if providing educational videos about atrial fibrillation to patients while they are waiting to see their doctor improves their knowledge and care experience.

3 What does participation in this research involve?
If you agree to participate, you will receive a survey that asks you some questions about your background, your knowledge of atrial fibrillation and your current medications. This survey will take approximately 5 minutes. You will then be randomly allocated to receive videos or not. There is a 50% chance you will receive videos and a 50% chance you will not. There are 4 videos and each of them last for approximately 4 minutes. After your clinic visit, we ask that you complete the surveys about atrial fibrillation knowledge and medications again. Our study coordinators will call you in 3 months to repeat the knowledge and medication questions with you. All videos required as part of the research project will be provided to you free of charge.

4 Can I have other treatments during this research project?
You do not have to take part in this research project to receive treatment at this hospital. Whilst you are participating in this research project, you are able to take all of the medications or treatments you have been taking for your condition or for other reasons.

5 What if I withdraw from this research project?
If you decide to withdraw from the project, please notify a member of the research team before doing so. Additional personal information will not collected from you. However, personal information already collected will be retained to ensure that the results of the research project can be measured properly and to comply with the law.

6 What happens when the research project ends?
Study results will be reported in peer-reviewed papers and on our institutional websites. In any publication and/or presentation about this research, information will be de-identified. All results will not contain your name and will be presented as a group, not as individuals. We will also email participants a summary of study results in English.

7 What will happen to information about me?
Any information obtained in connection with this research project that can identify you will remain confidential. Your name will not be included in study databases, and all information will

be stored using a study code. Your information will only be used for the purpose of this research project and it will only be disclosed with your permission, except as required by law.

In accordance with relevant Australian and NSW privacy and other relevant laws, you have the right to request access to your information collected and stored by the research team. You also have the right to request that any information with which you disagree be corrected. Please contact the study team member named at the end of this document if you would like to access your information.

8 Who is organising and funding the research?
This research project is being conducted by Dr Aravinda Thiagalingam and is funded by the Research and Education Grant Scheme. No member of the research team will receive a personal financial benefit from your involvement in this research project (other than their ordinary wages).

9 Who has reviewed the research project?
All research in Australia involving humans is reviewed by an independent group of people called a Human Research Ethics Committee (HREC). The ethical aspects of this research project have been approved by the HREC of Western Sydney Local Health District. This project will be carried out according to the *National Statement on Ethical Conduct in Human Research (2007)*. This statement has been developed to protect the interests of people who agree to participate in human research studies.

10 Further information and who to contact
If you want any further information concerning this project you can contact the principal study doctor Dr Aravinda Thiagalingam on 98456795 or any of the following people:

Clinical contact person

Name	*Dr Aravinda Thiagalingam*
Position	*Principal Investigator*
Telephone	*98456795*
Email	*aravinda.thiagalingam@sydney.edu.au*

For matters relating to research at the site at which you are participating, the details of the local site complaints person are:

Complaints contact person

Position	*Patient Advice and Liaison Service, Westmead Hospital*
Telephone	*8890 7014*
Email	wslhd-pals-mail@health.nsw.gov.au

If you have any complaints about any aspect of the project, the way it is being conducted or any questions about being a research participant in general, then you may contact:

Reviewing HREC approving this research and HREC Executive Officer details

Reviewing HREC name	*Western Sydney Local Health District*
HREC Executive Officer	*Kellie Hansen*
Telephone	8890 9007
Email	*Wslhd-researchoffice@health.nsw.gov.au*

Local HREC Office contact (Single Site -Research Governance Officer)

Position	*Research Governance Officer*
Telephone	*8890 9007*
Email	wslhd-researchoffice@health.nsw.gov.au

By clicking yes, you are agreeing to the following declaration:

- I have read the Participant Information Sheet or someone has read it to me in a language that I understand.

- I understand the purposes, procedures and risks of the research described in the project.

- I give permission for my doctors, other health professionals, hospitals or laboratories outside this hospital to release information to Westmead Hospital concerning your disease and treatment for the purposes of this project. I understand that such information will remain confidential.

- I acknowledge that any regulatory authorities may have access to my medical records specifically related to this project to monitor the research in which I am agreeing to participate. However, I understand my identity will not be disclosed to anyone else or in publications or presentations.

- I have had an opportunity to ask questions and I am satisfied with the answers I have received.

- I freely agree to participate in this research project as described and understand that I am free to withdraw at any time during the study without affecting my future health care.

- I understand that I will be given a signed copy of this document to keep.

- I give permission for study coordinators to call me in 3 months to fill a 5 minute survey

Today's date 06-05-2020 Today D-M-Y

* must provide value

Next Page >>

Resize font:

THE UNIVERSITY OF SYDNEY

Westmead Applied Research Centre

Informed Consent

Displayed below is a read-only copy of your survey responses. Please review it and the options at the bottom.

☑ I certify that all the information in the document above is correct. I understand that clicking 'Submit' will electronically sign the form and that signing this form electronically is the equivalent of signing a physical document.

If any information above is not correct, you may click the 'Previous Page' button to go back and correct it.

<< Previous Page

Submit

11. eReferences:

1. Rahman, F., Kwan, G.F. & Benjamin, E.J. Global epidemiology of atrial fibrillation. *Nature Reviews Cardiology* **11**, 639-654 (2014).
2. Kirchhof, P., *et al.* 2016 ESC Guidelines for the management of atrial fibrillation developed in collaboration with EACTS. *European Heart Journal* **37**, 2893-2962 (2016).
3. Brieger, D., *et al.* National Heart Foundation of Australia and the Cardiac Society of Australia and New Zealand: Australian Clinical Guidelines for the Diagnosis and Management of Atrial Fibrillation 2018. *Heart, Lung and Circulation* **27**, 1209-1266 (2018).
4. Guo, Y., *et al.* Mobile Health Technology for Atrial Fibrillation Management Integrating Decision Support, Education, and Patient Involvement: mAF App Trial. *The American Journal of Medicine* **130**, 1388-1396.e1386 (2017).
5. Hendriks, J.M., *et al.* Home-Based Education and Learning Program for Atrial Fibrillation: Rationale and Design of the HELP-AF Study. *Canadian Journal of Cardiology* **35**, 846-854 (2019).
6. Harris, P.A., *et al.* Research electronic data capture (REDCap)--a metadata-driven methodology and workflow process for providing translational research informatics support. *J Biomed Inform* **42**, 377-381 (2009).
7. Harris, P.A., *et al.* Research electronic data capture (REDCap)—A metadata-driven methodology and workflow process for providing translational research informatics support. *Journal of Biomedical Informatics* **42**, 377-381 (2009).
8. Cutter, G.R., *et al.* Cardiovascular risk factors in young adults. The CARDIA baseline monograph. *Control Clin Trials* **12**, 1s-77s (1991).
9. Gehi, A., Haas, D., Pipkin, S. & Whooley, M.A. Depression and Medication Adherence in Outpatients With Coronary Heart Disease: Findings From the Heart and Soul Study. *Archives of internal medicine* **165**, 2508-2513 (2005).
10. Reading, S.R., *et al.* Risk factors for medication non-adherence among atrial fibrillation patients. *BMC cardiovascular disorders* **19**, 38 (2019).
11. Desteghe, L., *et al.* Knowledge gaps in patients with atrial fibrillation revealed by a new validated knowledge questionnaire. *International journal of cardiology* **223**, 906-914 (2016).
12. McNaughton, C.D., *et al.* Health literacy and mortality: a cohort study of patients hospitalized for acute heart failure. *Journal of the American Heart Association* **4**(2015).

eAppendix 2: Statistical analysis plan

1. Synopsis

Atrial fibrillation (AF) is the most common cardiac arrhythmia, with prevalence expected to double by 2050.[13] Management of AF is complex, requiring patient adherence to potentially 3 classes of medication (rate/rhythm control, anticoagulation) and lifestyle modification. Patient education is a key component of holistic AF management, however there is limited availability of evidence-based education that is relevant to patients in Australian contexts.[3] Clinician-created education may provide a solution to bridge this gap but there are few examples of randomised studies assessing its efficacy in improving patient knowledge, adherence, or satisfaction with clinical care.

This study aims to investigate in a randomised clinical trial (RCT), among 200 patients in inpatient and outpatient hospital settings with AF, the impact of continued exposure to a series of clinician-created educational videos on patient knowledge of AF at 90 days follow up compared to usual care. Key secondary outcomes will include satisfaction with clinical care, motivation to maintain medication adherence, and medication adherence.

1.1 Primary objective

To examine, in a randomised controlled trial of 200 hospital inpatients and outpatients with AF, the impact of continued exposure to clinician-created educational videos on AF knowledge at 90 day follow up.

1.2 Secondary objectives

To investigate whether, when compared to usual care, patients who are exposed to clinician-created videos are:

- More satisfied with their clinical care
    - Highly satisfied - ≥ 6/7 on Likert scale, not highly satisfied ≤ 5/7
- More motivated to adhere to AF medications at 2 and 90 days
    - Highly motivated - ≥ 6/7 on Likert scale, not highly motivated ≤ 5/7
- More adherent to medications at 90 days (see appendix 1: medication adherence questionnaire)

Non-adherence will be defined as

4. Response to question 1 of “Most of the time (75%) or less OR
5. Response to question 2 of “once per week” or more OR
6. Response to question 3 of “once per week” or more OR

1.3 Trial design

Randomised clinical trial with randomisation to intervention or control with ratio 1:1 respectively (n=200, intervention = 100, control = 100). Intervention participants will receive educational videos on top of usual care and control participants will receive usual care.

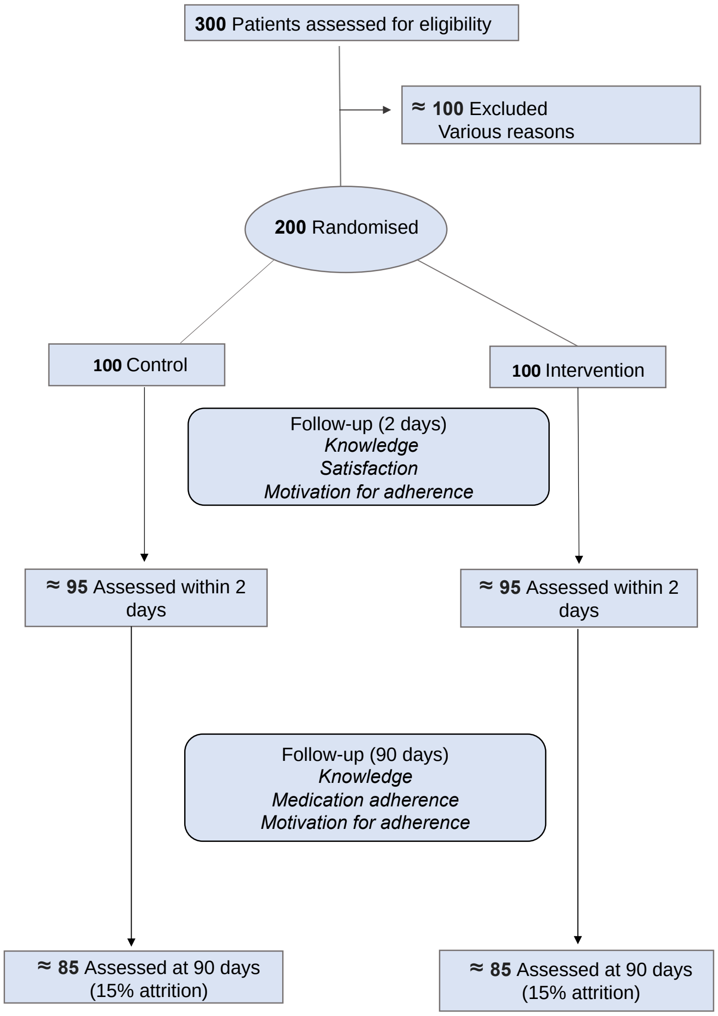

eFigure 1: Participant recruitment and follow up

1.4 Interventions

Patients randomised to the intervention arm will, following completion of remote consent, receive access to a series of 4 educational videos describing AF, its pathophysiology, pharmacological management, and lifestyle modification.

1.5 Subgroups

The interaction of treatment effect and age, gender, education, ethnicity, subtype of AF and category of presentation will be explored but the study is not powered for these subgroups.

1.6 Power and sample size

A sample size of 200 participants (100 per arm) would, allowing for 15% attrition and assuming a standard deviation of 18 as described in other studies,[11] have 90% power (2 sided, type 1 error of 5%) to detect an difference in 90-day AF knowledge of 8.95% between participants in the intervention and control arms of the study, which we consider a significant improvement in knowledge. Previous assessment of the JAFKQ has indicated participant scores 1-month post-test with no educational intervention are unchanged (60.4 [SD = 18.6] vs 62.3 [SD = 18.5]; p = 0.551).[11,14,15] Analysis will follow intention to treat principles with all participants analysed in the arm they have been randomised to. P-values of < 0.05 will be considered significant. Primary outcome analysis will use a two-tailed t-test to assess the difference between mean participant JAFKQ scores (max score = 100%). Analysis will also be performed utilizing an Analysis of Covariance (ANCOVA), with baseline JAFKQ scores and education level incorporated into the model as covariates.

For secondary analysis of satisfaction with clinical care, patient responses will be dichotomised into 'highly satisfied' (≥ 6/7) vs 'not highly satisfied'(≤ 5/7). For medication adherence, patients will be classified as "adherent" or "not adherent". Proportions in intervention vs control arms will be compared using a chi-squared test. Relative risk will be calculated using a log binomial unadjusted and adjusted for total time in clinic (for outpatients) and number of medications. Allowing for 15% attrition and assuming 60% satisfaction/medium or greater adherence in the control groups, a sample size of 200 (100 per arm) would have 90% power to detect a relative risk difference in satisfaction and adherence between control and intervention groups of 1.37 (that is, an increase from 60.0% to 82.2%).

1.7 Schedule of Data Collection

eTable 1: Schedule of outcomes to be assessed

Survey	Baseline	Post-clinic/within 2 days of intervention delivery	90-day follow up
Baseline demographic information	X		
Satisfaction with care (7 – point Likert scale)		X	
Medication adherence	X		X
Motivation to maintain adherence to AF therapy (7-point Likert scale)	X	X	X
AF Knowledge (Jessa AF Knowledge Questionnaire)	X	X	X

2. Patient Population

The study population will be patients with confirmed AF on an electrocardiogram presenting to Westmead Hospital for clinical care. This includes both inpatients and outpatients.

2.1 Inclusion criterion

Inclusion

5. Adults aged 18 years and over
6. Atrial fibrillation or atrial flutter diagnosed on electrocardiogram

7. $CHA_2DS_2VASc > 0$ and/or currently prescribed anticoagulation therapy
8. Have an email address or active mobile number

Exclusion

4. Too unwell (physically or mentally) to participate in surveys
5. Previously allocated to either condition of the study
6. Insufficient English to understand video content or to participate in the audit surveys

3. Analysis

3.1 Analysis Principles

General methodology

All statistical tests will be two-tailed, and a 5% significance level adopted throughout.
Analyses will be performed according to the principal of intention to treat and will include all randomised patients.
Summaries of continuous baseline variables will be presented as means and standard deviations together with medians and inter-quartile ranges. Categorical variables will be presented as frequencies and percentages. All continuous data will be checked for normality
Methods for handling missing data are described in 3.2 – sensitivity analysis. If 80% of survey data is available, missing data will be imputed. Participants with less than 80% of outcome data available for a given survey will be removed from analysis.
Baseline values of the analysed parameter will be used as a covariate for analysis that support covariates, if these are not available see approach described below.
Balance in baseline risk factors will be considered between intervention and control groups, and if significant differences exist, additional regression analysis of outcome variables adjusting for this difference will be performed.

Primary outcome analysis:

The primary outcome will be analysed utilising an analysis covariance (ANCOVA), with age, gender, education and subtype of AF incorporated as covariates. Where baseline data are available, this will also be incorporated into the model as a covariate. An unadjusted two-tailed t-test will also be performed. We will report p-values and 95% confidence intervals.

Secondary outcome analysis:

The significance of any difference between proportional secondary outcomes (satisfaction with clinical care, motivation to maintain adherence, actual medication adherence) will be analysed using a log binomial model adjusting for age (continuous), gender (male vs female), education (year 11 and above vs less than year 11), sub-type of AF (categorical). A relative risk, 95% confidence interval and p-value will also be reported. Where baseline results are available, the corresponding baseline value results (e.g. discrete total score) will also be included as a covariate. Where this does not converge, a robust poisson will be fitted.
An unadjusted chi squared test will also be calculated with a corresponding relative risk, 95% confidence interval and p-value.

3.2 Sensitivity Analysis

If there are more than 10% of values missing for any primary or secondary outcome, a sensitivity analysis will be conducted using a logistic regression with the outcome being missing or not and exploring any associations with demographic and medical baseline variables to understand any biases that the missingness may introduce.

3.3 As Treated Analyses

We will also perform an as treated analysis examining all primary and secondary outcome variables with respect to their engagement with the study intervention. Highly engaged participants will be those that watched study videos on 3 or more occasions, moderately engaged participants those who watched study videos on 1-2 occasions and poorly engaged participants those who did not watch study videos.

3.4 Summary of Patient Disposition

eTable 2: Summary of Patients Disposition

	**Control** **N_C (%)**	**Intervention (Toal)** **N_T(%)**

**Patients randomised**	n/N_C (%)	n/N_T (%)
**Lost to follow-up at 2 days**	n/N_C (%)	n/N_T (%)
**Lost to follow up at 90 days**	n/N_C (%)	n/N_T (%)
**Withdrew consent***	n/N_C (%)	n/N_T (%)

*Details on reasons for consent withdrawal will be summarised as shown in etable 3

eTable 3: Summary of Withdrawal Reasons

**Patient Id**	**Treatment group**	**Discontinued/ withdrawn consent**	**Reason**
2	*Intervention*	*Discontinued*	
111	*Control*	*Discontinued*	
300	*Intervention*	*Withdrawn consent*	

3.5 Data Completeness and distribution

3.5.1 Data available for endpoint analysis

All the data available for endpoint analysis will be presented as shown in etable 4.

eTable 4: Data completeness for endpoint analysis

**Outcome**	**Control N_C**	**Intervention (Total) N_T**	**Total N**
**Overall Survey completeness**			
**Baseline Survey**			
Inside window	N=x (%)	N=x (%)	N=x (%)
Outside window	N=x (%)	N=x (%)	N=x (%)
Missing	N=x (%)	N=x (%)	N=x (%)
**2 day surveys**			
Inside window	N=x (%)	N=x (%)	N=x (%)
Outside window	N=x (%)	N=x (%)	N=x (%)
Missing	N=x (%)	N=x (%)	N=x (%)
**90 day surveys**			
Inside window	N=x (%)	N=x (%)	N=x (%)
Outside window	N=x (%)	N=x (%)	N=x (%)
Missing	N=x (%)	N=x (%)	N=x (%)

Inside window = 2 day survey completed within 7 days, 90-day survey completed within 120 days. Complete is defined if >80% of a survey is complete.

3.5.2 Data available for baseline demographic and medical history analysis

In addition, data available for baseline demographic and medical status will be presented as shown in etable 5.

eTable 5: Baseline data completeness – Medical characteristics

Baseline characteristic	Control NP (%)	Intervention (Total) N(%)	Total N(%)
**Medical History**			
**Sub-type of AF**			
**Atrial fibrillation**	N=x (%)	N=x (%)	N=x (%)
**Atrial flutter**	N=x (%)	N=x (%)	N=x (%)
**Valvular vs non-valvular**			
**Valvular**	N=x (%)	N=x (%)	N=x (%)
**Non-valvular**	N=x (%)	N=x (%)	N=x (%)
**Length of AF**			
**First AF episode**	N=x (%)	N=x (%)	N=x (%)
**Paroxysmal AF**	N=x (%)	N=x (%)	N=x (%)
**Permanent AF**	N=x (%)	N=x (%)	N=x (%)
**Predominant flutter**	N=x (%)	N=x (%)	N=x (%)
**Time since AF diagnosis**	N=x (%)	N=x (%)	N=x (%)
**Comorbidities**			
**Diabetic status**	N=x (%)	N=x (%)	N=x (%)
**Hyperlipidaemia**	N=x (%)	N=x (%)	N=x (%)

**Hypertension**	N=x (%)	N=x (%)	N=x (%)
**Existing coronary heart disease[1]**	N=x (%)	N=x (%)	N=x (%)
**Previous stroke**	N=x (%)	N=x (%)	N=x (%)
**Peripheral vascular disease**	N=x (%)	N=x (%)	N=x (%)
**Chronic kidney disease**	N=x (%)	N=x (%)	N=x (%)
**Number of current medications**	N=x (%)	N=x (%)	N=x (%)
**Motivated to maintain medication adherence**	N=x (%)	N=x (%)	N=x (%)
**Adherent to medications**	N=x (%)	N=x (%)	N=x (%)

[1]CHD defined as one of the following: Prior myocardial infarction, coronary artery bypass graft surgery, percutaneous coronary intervention, or 50% or greater stenosis in at least 1 major epicardial vessel on coronary angiography.

3.5.3 Baseline demographic and medical maximum/minimum values

Maximum and minimum values for baseline demographic and medical characteristics will be presented as shown in etable 6. As all other variables are categorical, max/min values will only be displayed for Age, total time in clinic, and clinical assessment.

eTable 6: Demographic maximum/minimum values

Baseline Characteristics	Control N_C (%)	Intervention (Total) N_T(%)	Total N(%)
**Age**	Min=X, Max=X	Min=X, Max=X	Min=X, Max=X

3.6 Patient Baseline Characteristics

Patient baseline demographic, medical and lifestyle characteristics will be displayed as shown below in etables 7 and 8. We will also describe the characteristics of the clinic setting and intervention delivery – including total time spent in clinic, number of videos watched, total time watching videos. Results will be tabulated and/or displayed in frequency histograms or bar graphs to demonstrate distribution.

eTable 7: Baseline Demographic and Lifestyle Characteristics by Treatment Group

Baseline Characteristics	Control N_C (%)	Intervention (Total) N_T(%)	Total N(%)
**Male**	n/N_C (%)	n/N_T (%)	n/N (%)
**Age**	(n=xx)<br>Mean (SD)<br>Median (IQR)	(n=xx)<br>Mean (SD)<br>Median (IQR)	(n=xx)<br>Mean (SD)<br>Median (IQR)
**Ethnicity**			
**Aboriginal/Torres Strait Isldr**	n/N_C (%)	n/N_T (%)	n/N (%)
**European**	n/N_C (%)	n/N_T (%)	n/N (%)

**Asian (North, East, South-East)**	n/N_C (%)	n/N_T (%)	n/N (%)
**Middle-East and North African**	n/N_C (%)	n/N_T (%)	n/N (%)
**Other**	n/N_C (%)	n/N_T (%)	n/N (%)
**Educational level**			
**Did not attend school**	n/N_C (%)	n/N_T (%)	n/N (%)
**Attended school, did not complete 12 or equivalent**	n/N_C (%)	n/N_T (%)	n/N (%)
**Completed tertiary qualification (Trade certificate, diploma, bachelor or post-graduate study)**	n/N_C (%)	n/N_T (%)	n/N (%)
**Number of Videos Watched**		(n=xx)<br>Mean (SD)<br>Median (IQR)	

eTable 8: Baseline Medical Characteristics by treatment group

**Baseline characteristic**	**Control NP (%)**	**Intervention (Total) N(%)**	**Total N(%)**
**Medical History**			
**Sub-type of AF**			
**Atrial fibrillation**	n/N_C (%)	n/N_T (%)	n/N (%)
**Atrial flutter**	n/N_C (%)	n/N_T (%)	n/N (%)
**Valvular vs non-valvular**			
**Valvular**	n/N_C (%)	n/N_T (%)	n/N (%)
**Non-valvular**	n/N_C (%)	n/N_T (%)	n/N (%)
**Length of AF**			
**First AF episode**	n/N_C (%)	n/N_T (%)	n/N (%)
**Paroxysmal AF**	n/N_C (%)	n/N_T (%)	n/N (%)
**Permanent AF**	n/N_C (%)	n/N_T (%)	n/N (%)

**Predominant flutter**	n/N_C (%)	n/N_T (%)	n/N (%)
**Time since AF diagnosis**	Median (IQR)	Median (IQR)	Median (IQR)
**Comorbidities**			
**Diabetic status**	n/N_C (%)	n/N_T (%)	n/N (%)
**Hyperlipidaemia**	n/N_C (%)	n/N_T (%)	n/N (%)
**Hypertension**	n/N_C (%)	n/N_T (%)	n/N (%)
**Existing coronary heart disease[1]**	n/N_C (%)	n/N_T (%)	n/N (%)
**Previous stroke**	n/N_C (%)	n/N_T (%)	n/N (%)
**Peripheral vascular disease**			
**Chronic kidney disease**	n/N_C (%)	n/N_T (%)	n/N (%)
**Number of current medications**	Median (IQR)	Median (IQR)	Median (IQR)
**Motivated to maintain medication adherence**	n/N_C (%)	n/N_T (%)	n/N (%)
**Motivated to maintain medication adherence**	n/N_C (%)	n/N_T (%)	n/N (%)

[1]CHD defined as one of the following: Prior myocardial infarction, coronary artery bypass graft surgery, percutaneous coronary intervention, or 50% or greater stenosis in at least 1 major epicardial vessel on coronary angiography.

4 Primary Outcome – Participant AF knowledge at 90 days

4.1 Primary Outcome Determination

The primary outcome of this study is knowledge of AF medications at 90 day follow up.
This will be defined as the overall score for participants (out of 100%) for participants on the Jessa Atrial Fibrillation Knowledge Questionnaire (JAFKQ).

4.1.1 Primary and key secondary outcome Analysis

The primary outcome will be analysed utilising an analysis of covariance (ANCOVA) comparing scores between intervention and control groups and adjusting for age, gender, educational level and sub-type of AF.
The primary outcome will also be analysed utilising a two tailed t-test comparing the mean score on the JAFKQ between intervention and control participants. Primary and secondary analysis is described in etable 9.

eTable 9: Primary and key secondary outcome analysis

	**Control N_C (%)**	**Intervention N_T(%)**	**RR [95% CI] (Control vs CPR video) [unadjusted]**	**RR [95% CI] (Control vs CPR video) [adjusted]**	**p-value [adjusted]**
**Jessa Atrial Fibrillation Knowledge Questionnaire score (%)**					
Mean score 2 days post intervention	(n=xx)<br>Mean (SD)<br>Median (IQR)	(n=xx)<br>Mean (SD)<br>Median (IQR)		Mean Difference (95% CI)	p-value

Mean score 90 days post intervention	(n=xx) Mean (SD) Median (IQR)	(n=xx) Mean (SD) Median (IQR)		Mean Difference (95% CI)	p-value
**Satisfaction**					
Highly satisfied*	(n=xx) Proportion (%)	(n=xx) Proportion (%)		RR (95% CI)	p-value
**Motivation to maintain medication adherence**					
Highly motivated* 2 days post intervention	(n=xx) Proportion (%)	(n=xx) Proportion (%)		RR (95% CI)	p-value
Highly motivated* 90 days post intervention	(n=xx) Proportion (%)	(n=xx) Proportion (%)		RR (95% CI)	p-value
**Medication adherence**					
Adherent** to medications 90 days post intervention	(n=xx) Proportion (%)	(n=xx) Proportion (%)		RR (95% CI)	p-value

**Highly motivated/satisfied = Reported motivation to maintain medication adherence/satisfaction with clinical care ≥ 6 on a 7-point likert scale*

5 Secondary outcome analysis

5.1 Satisfaction with clinic experience, motivation to maintain medication adherence and medication adherence – outcome determination

Satisfaction with clinic experience will be determined by a score of ≥ 6 on a 7-point Likert scale. The proportion of participants achieving this outcome will be compared between intervention and control groups.
Motivation to maintain medication adherence will be determined by a score of ≥ 6 on a 7-point Likert scale. The proportion of participants achieving this outcome will be compared between intervention and control groups.
Medication adherence will be determined by responses to the following questions (previously validated for patients with coronary artery disease and utilised in large scale studies of patients with AF):

4) **Specific to your atrial fibrillation medication, in the past month, how often did you take your medications as the doctor prescribed?**
    - **a.** All of the time (100%)
    - **b.** Nearly all of the time (90%)
    - **c.** Most of the time (75%)
    - **d.** About half the time (50%)
    - **e.** Less than half the time (<50%)
    - **f.** No atrial fibrillation medication

5) **Specific to your atrial fibrillation medication, in the past month, how often did you forget to take one or more of your prescribed medications?**
    - **a.** Never
    - **b.** Once
    - **c.** 2-3 times
    - **d.** Once per week
    - **e.** Several times per week
    - **f.** Nearly every day
    - **g.** No atrial fibrillation medicaiton

6) **Specific to your atrial fibrillation medication, in the past month, how often did you decide to skip one or more of your prescribed medications**

a. Never
b. Once
c. 2-3 times
d. Once per week
e. Several times per week
f. Nearly every day
g. No atrial fibrillation medicaiton

Non-adherence will be defined as per previous studies:

7. Response to question 1 of "Most of the time (75%) or less OR
8. Response to question 2 of "once per week" or more OR
9. Response to question 3 of "once per week" or more OR

5.1.1 Satisfaction with clinic, motivation to maintain medication adherence and medication adherence – Analysis

Key secondary outcomes (satisfaction, motivation to maintain medication adherence and medication adherence) will be assessed utilising a log binomial adjusting for age, gender, educational level and sub-type of AF. This analysis is encompassed within etable 9.
An unadjusted chi-squared test will also be performed comparing these two outcomes.

5.1.2 As treated analysis of primary and secondary outcomes:

An as treated analysis of all primary and secondary outcomes will also be performed to assess the impact of intervention engagement on study outcomes.

Highly engaged participants will be defined as those watching the video series 3 or more times throughout the intervention period. Moderately engaged participants those watching the series 1-2 times, and poorly engaged participants those who did not watch the video series. Each intervention group will be compared to the control group in the same analyses described above (etables 10-12).

eTable 10: Sub-group analysis of poorly engaged intervention participants

	Control	Poorly	RR [95% CI]	RR [95% CI]	p-value

	N_C (%)	engaged Intervention N_T(%)	(Control vs CPR video) [unadjusted]	(Control vs CPR video)	[adjusted]
**Jessa Atrial Fibrillation Knowledge Questionnaire score (%)**					
Mean score 2 days post intervention	(n=xx) Mean (SD) Median (IQR)	(n=xx) Mean (SD) Median (IQR)		Mean Difference (95% CI)	p-value
Mean score 90 days post intervention	(n=xx) Mean (SD) Median (IQR)	(n=xx) Mean (SD) Median (IQR)		Mean Difference (95% CI)	p-value
**Satisfaction**					
Highly satisfied*	(n=xx) Proportion (%)	(n=xx) Proportion (%)		RR (95% CI)	p-value
**Motivation to maintain medication adherence**					
Highly motivated* 2 days post intervention	(n=xx) Proportion (%)	(n=xx) Proportion (%)		RR (95% CI)	p-value
Highly motivated* 90 days post intervention	(n=xx) Proportion (%)	(n=xx) Proportion (%)		RR (95% CI)	p-value
**Medication adherence**					
Adherent** to medications 90 days post intervention	(n=xx) Proportion (%)	(n=xx) Proportion (%)		RR (95% CI)	p-value

eTable 11: Sub-group analysis of moderately engaged intervention participants

	**Control N_C (%)**	**Moderately engaged Intervention N_T(%)**	**RR [95% CI] (Control vs CPR video)**	**p-value**
**Jessa Atrial Fibrillation Knowledge Questionnaire score (%)**				
Mean score 2 days post intervention	(n=xx) Mean (SD) Median (IQR)	(n=xx) Mean (SD) Median (IQR)	Mean Difference (95% CI)	p-value
Mean score 90 days post intervention	(n=xx) Mean (SD) Median (IQR)	(n=xx) Mean (SD) Median (IQR)	Mean Difference (95% CI)	p-value
**Satisfaction**				
Highly satisfied*	(n=xx) Proportion (%)	(n=xx) Proportion (%)	RR (95% CI)	p-value
**Motivation to maintain medication adherence**				
Highly motivated* 2 days post intervention	(n=xx) Proportion (%)	(n=xx) Proportion (%)	RR (95% CI)	p-value
Highly motivated* 90 days post intervention	(n=xx) Proportion (%)	(n=xx) Proportion (%)	RR (95% CI)	p-value
**Medication adherence**				
Adherent** to medications 90 days post intervention	(n=xx) Proportion (%)	(n=xx) Proportion (%)	RR (95% CI)	p-value

eTable 12: Sub-group analysis of highly engaged intervention participants

	**Control N_C (%)**	**Highly engaged Intervention N_T(%)**	**RR [95% CI] (Control vs CPR video)**	**p-value**
**Jessa Atrial Fibrillation Knowledge Questionnaire score (%)**				
Mean score 2 days post intervention	(n=xx)<br>Mean (SD)<br>Median (IQR)	(n=xx)<br>Mean (SD)<br>Median (IQR)	Mean Difference (95% CI)	p-value
Mean score 90 days post intervention	(n=xx)<br>Mean (SD)<br>Median (IQR)	(n=xx)<br>Mean (SD)<br>Median (IQR)	Mean Difference (95% CI)	p-value
**Satisfaction**				
Highly satisfied*	(n=xx)<br>Proportion (%)	(n=xx)<br>Proportion (%)	RR (95% CI)	p-value
**Motivation to maintain medication adherence**				
Highly motivated* 2 days post intervention	(n=xx)<br>Proportion (%)	(n=xx)<br>Proportion (%)	RR (95% CI)	p-value
Highly motivated* 90 days post intervention	(n=xx)<br>Proportion (%)	(n=xx)<br>Proportion (%)	RR (95% CI)	p-value
**Medication adherence**				
Adherent** to medications 90 days post intervention	(n=xx)<br>Proportion (%)	(n=xx)<br>Proportion (%)	RR (95% CI)	p-value

6. eReferences

1. Rahman, F., Kwan, G.F. & Benjamin, E.J. Global epidemiology of atrial fibrillation. *Nature Reviews Cardiology* **11**, 639-654 (2014).
2. Kirchhof, P., *et al.* 2016 ESC Guidelines for the management of atrial fibrillation developed in collaboration with EACTS. *European Heart Journal* **37**, 2893-2962 (2016).
3. Brieger, D., *et al.* National Heart Foundation of Australia and the Cardiac Society of Australia and New Zealand: Australian Clinical Guidelines for the Diagnosis and Management of Atrial Fibrillation 2018. *Heart, Lung and Circulation* **27**, 1209-1266 (2018).
4. Guo, Y., *et al.* Mobile Health Technology for Atrial Fibrillation Management Integrating Decision Support, Education, and Patient Involvement: mAF App Trial. *The American Journal of Medicine* **130**, 1388-1396.e1386 (2017).
5. Hendriks, J.M., *et al.* Home-Based Education and Learning Program for Atrial Fibrillation: Rationale and Design of the HELP-AF Study. *Canadian Journal of Cardiology* **35**, 846-854 (2019).
6. Harris, P.A., *et al.* Research electronic data capture (REDCap)--a metadata-driven methodology and workflow process for providing translational research informatics support. *J Biomed Inform* **42**, 377-381 (2009).
7. Harris, P.A., *et al.* Research electronic data capture (REDCap)—A metadata-driven methodology and workflow process for providing translational research informatics support. *Journal of Biomedical Informatics* **42**, 377-381 (2009).
8. Cutter, G.R., *et al.* Cardiovascular risk factors in young adults. The CARDIA baseline monograph. *Control Clin Trials* **12**, 1s-77s (1991).
9. Gehi, A., Haas, D., Pipkin, S. & Whooley, M.A. Depression and Medication Adherence in Outpatients With Coronary Heart Disease: Findings From the Heart and Soul Study. *Archives of internal medicine* **165**, 2508-2513 (2005).
10. Reading, S.R., *et al.* Risk factors for medication non-adherence among atrial fibrillation patients. *BMC cardiovascular disorders* **19**, 38 (2019).
11. Desteghe, L., *et al.* Knowledge gaps in patients with atrial fibrillation revealed by a new validated knowledge questionnaire. *Int J Cardiol* **223**, 906-914 (2016).
12. McNaughton, C.D., *et al.* Health literacy and mortality: a cohort study of patients hospitalized for acute heart failure. *Journal of the American Heart Association* **4**(2015).
13. Kornej, J., Börschel, C.S., Benjamin, E.J. & Schnabel, R.B. Epidemiology of Atrial Fibrillation in the 21st Century. *Circulation Research* **127**, 4-20 (2020).
14. Desteghe, L., *et al.* Effectiveness and usability of an online tailored education platform for atrial fibrillation patients undergoing a direct current cardioversion or pulmonary vein isolation. *International journal of cardiology* **272**, 123-129 (2018).
15. Desteghe, L., *et al.* Effect of reinforced, targeted in-person education using the Jessa Atrial fibrillation Knowledge Questionnaire in patients with atrial fibrillation: A randomized controlled trial. *European journal of cardiovascular nursing : journal of the Working Group on Cardiovascular Nursing of the European Society of Cardiology* **18**, 194-203 (2019).
