# Supplementary material for: Clinician-Created Video Education for Patients With AF: A Randomized Clinical Trial
Source: JAMA Netw Open. 2023 Dec 8;6(12):e2345308. doi: 10.1001/jamanetworkopen.2023.45308 (PMC10709770; doi:10.1001/jamanetworkopen.2023.45308)
Supplement: Supplement 2. — eTable. Baseline Demographic Data by Completeness [file jamanetwopen-e2345308-s002.pdf]

Supplemental Online Content

McIntyre D, Kovoor JG, Chow CK, et al. Clinician-created video education for patients with AF. *JAMA Netw Open*. 2023;6(12):e2345308. doi:10.1001/jamanetworkopen.2023.45308

eTable. Baseline Demographic Data by Completeness

This supplemental material has been provided by the authors to give readers additional information about their work.

© 2023 McIntyre D et al. *JAMA Network Open.*

eTable: Baseline demographic data by completeness

Characteristic	**Did not complete baseline, 2 day or 90 day follow up, N = 27**		**Completed follow up at all 3 timepoints, N = 181**	
Age, mean (SD[1])	68 (13.5)		65 (12.1)	
Male (n %)	19 (82.6%)		114 (63.0%)	
**Ethnicity**				
Aboriginal/Torres strait Is	0 (0.0%)		1 (0.6%)	
Asian (North/East/South-East)	3 (13.0%)		20 (11.0%)	
Caucasian	11 (47.8%)		117 (64.6%)	
Middle-East and North African	2 (8.7%)		5 (2.8%)	
Other	7 (30.4%)		38 (21.0%)	
**Education**				
Completed year 12 (n %)	13 (56.5%)		106 (58.6%)	
Number videos watched, median (IQR[2])	0 (0, 0)		0 (0, 7)	
**Engagement**				
Poor (0 additional sessions)	21 (78%)		92 (51%)	
High (3 or more sessions)	-		44 (42.3%)	
Type of AF				
First episode AF	4 (17%)		9 (5.0%)	
Paroxysmal AF	13 (54%)		101 (56%)	
Permanent AF	7 (29%)		54 (30%)	
Predominant flutter	0 (0%)		15 (8.4%)	
Valvular vs non-valvular				
Valvular	4 (17%)		31 (17%)	
Comorbidities				
Diabetes	11 (46%)		27 (15%)	
Coronary artery disease	9 (38%)		43 (24%)	
Hypertension	15 (63%)		110 (61%)	
Stroke	0 (0%)		14 (7.8%)	
Peripheral vascular disease	0 (0%)		9 (5.1%)	
Chronic kidney disease	8 (33%)		21 (12%)	
**Current medications**				
1-4	6 (22.2%)		76 (42.0%)	
5-8	10 (37.0%)		71 (39.2%)	
More than 8	8 (29.6%)		19 (18.6%)	
**JAFKQ Scores**	*Control*	*Intervention*	*Control*	*Intervention*
Proportion (SD)	0.65 (0.20)	0.52 (0.23)	0.71 (0.18)	0.69 (0.19)

([1]SD = standard deviation, [2]IQR = Interquartile range)

© 2023 McIntyre D et al. *JAMA Network Open.*
