# Supplementary material for: Clinician-Created Video Education for Patients With AF: A Randomized Clinical Trial
Source: JAMA Netw Open. 2023 Dec 8;6(12):e2345308. doi: 10.1001/jamanetworkopen.2023.45308 (PMC10709770; doi:10.1001/jamanetworkopen.2023.45308)
Supplement: Supplement 3. — Data Sharing Statement [file jamanetwopen-e2345308-s003.pdf]

Data Sharing Statement

McIntyre. Clinician-Created Video Education for Patients With AF. *JAMA Netw Open*.
Published December 08, 2023. doi:10.1001/jamanetworkopen.2023.45308

Data
Data available: Yes
Data types: Deidentified participant data
How to access data: Data will be made available following assessment of a data use proposal submitted to investigators at daniel.mcintyre@sydney.edu.au.
When available: With publication

Supporting Documents
Document types: Informed consent form
How to access documents: This will be available in supplementary files.
When available: With publication

Additional Information
Who can access the data: Data will be made available to researchers whose proposed use of the data has been approved.
Types of analyses: For a specified purpose outlined in a data use proposal.
Mechanisms of data availability: After approval of a proposal and with a signed data access agreement.
